# Development and Validation of a Wearable Inertial Sensors-Based Automated System for Assessing Work-Related Musculoskeletal Disorders in the Workspace

**DOI:** 10.3390/ijerph17176050

**Published:** 2020-08-20

**Authors:** Chunxi Huang, Woojoo Kim, Yanxin Zhang, Shuping Xiong

**Affiliations:** 1 Human Factors and Ergonomics Laboratory, Department of Industrial and Systems Engineering, Korea Advanced Institute of Science and Technology (KAIST), 291 Daehak-ro, Yuseong-gu, Daejeon 34141, Korea; tracyhuang@kaist.ac.kr (C.H.); xml1324@kaist.ac.kr (W.K.); 2 Department of Exercise Sciences, The University of Auckland, 4703906, Newmarket, Auckland 1142, New Zealand; yanxin.zhang@auckland.ac.nz

**Keywords:** work-related musculoskeletal disorders, risk assessment, occupational safety, postural ergonomic analysis, static biomechanical analysis, system development and validation

## Abstract

The industrial societies face difficulty applying traditional work-related musculoskeletal disorder (WMSD) risk assessment methods in practical applications due to in-situ task dynamics, complex data processing, and the need of ergonomics professionals. This study aims to develop and validate a wearable inertial sensors-based automated system for assessing WMSD risks in the workspace conveniently, in order to enhance workspace safety and improve workers’ health. Both postural ergonomic analysis (RULA/REBA) and two-dimensional static biomechanical analysis were automatized as two toolboxes in the proposed system to provide comprehensive WMSD risk assessment based on the kinematic data acquired from wearable inertial sensors. The effectiveness of the developed system was validated through a follow-up experiment among 20 young subjects when performing representative tasks in the heavy industry. The RULA/REBA scores derived from our system achieved high consistency with experts’ ratings (intraclass correlation coefficient ≥0.83, classification accuracy >88%), and good agreement was also found between low-back compression force from the developed system and the reference system (mean intersystem coefficient of multiple correlation >0.89 and relative error <9.5%). These findings suggested that the wearable inertial sensors-based automated system could be effectively used for WMSD risk assessment of workers when performing tasks in the workspace.

## 1. Introduction

Work-related musculoskeletal disorders (WMSDs) have been recognized as a primary cause of non-fatal injuries and the main reason for absence from work in industrial societies for a long time [1]. Nowadays, WMSDs are a worldwide concern and distributed among both industrialized countries and industrially developing countries [2,3]. It is necessary to mitigate the WMSDs as they hurt the health profile of workers [4,5], reduce work productivity [6], and increase the medical and compensation cost [7].

To prevent WMSDs, assessing exposure to risk factors of WMSDs was proven to be one of the most effective approaches [8]. Much research effort has been spent to develop assessment methods and tools to identify the risk factors of WMSDs. Most of the WMSD assessment methods could be broadly classified into two categories: (1) postural ergonomic analysis for identifying awkward postures and other improper task settings and (2) biomechanical analysis for recognizing the potential musculoskeletal injuries resulting from biomechanical loading, such as massive internal forces and moments at the joints [9]. Throughout the development history of WMSD assessment methods, many self-report questionnaires such as the Nordic Musculoskeletal Questionnaire [10,11], Borg Scale [12,13], and Job Requirements and Physical Demands Survey (JRPDS) [14] were first introduced to assess musculoskeletal symptoms in the industrial environment. However, the results of self-report were based on subjective assessments, which could have high bias between individuals [15], especially for industry workers without any professional knowledge about WMSDs, thereby leading to low reliability of the results [16]. To overcome the limitations of self-report methods, some observational tools have been developed, such as the Ovako Working Posture Analyzing System (OWAS) [17], Posture Activity Tools and Handling (PATH) [18], Rapid Upper Limb Assessment (RULA) [19], and Rapid Entire Body Assessment (REBA) [20]. Even though observational methods are easy to use and require minimal instrumentation, they rely on ergonomics experts’ involvement [21], and the inter-rater variability may cause disagreement among the results from different experts [22,23]. With the technological advancements of the last 20 years, some objective methods (e.g., human motion capture [24,25,26], lumbar motion monitor [27], and electromyography [28]) have been used to assist or replace expert observation, which could help to improve the accuracy of WMSD risk assessment. Biomechanical methods have also been developed to assess the WMSD risk in terms of in-vivo joint contact force and moment, and muscle force [29,30,31].

Based on the WMSD risk assessment methods and the kinematic information acquired from scientific instrumentations, several studies attempted to develop automated WMSD assessment systems, which aimed to provide rapid, accurate, convenient, and expert-free WMSD risk assessment for enhancing workspace safety. For example, Seo et al. proposed an automated biomechanical analysis for assessing the risk of WMSDs using motion data obtained from an optical motion capture system [31,32]; however, this approach suffered from impracticality in the actual workspace due to the cumbersome setup and occlusion-sensitive nature of the optical motion capture systems [33]. Researchers also proposed automated WMSD risk assessment utilizing computer vision-based motion capture data as input for various methods such as OWAS [34], RULA [35], biomechanical modeling [36], biomechanical analysis [9], and joint-level workload evaluation [37]. Nevertheless, these methods were not free from concerns on applicability, majorly due to the low accuracy [38] typically found in a cluttered work environment with complex working postures. To overcome the aforementioned disadvantages of optical and vision-based motion capture, the wearable inertial sensors-based system [39,40,41] has been proposed as an alternative. The wearable inertial sensors-based approach was considered as a good compromise for enabling automated WMSD risk assessment in the complex workspace benefiting from its wearability and being easy to use in the field compared to optical-based approaches and high accuracy compared to vision-based approaches.

Surprisingly, the validation of these developed systems is severely lacking. Most studies have not validated their developed systems and only a few studies have conducted validation experiments but with simple experimental tasks, such as bending and scanning [40,41], which may not be sufficient to prove the reliability and robustness of the developed system given the complexity of various tasks of real work scenarios. The necessity of further systematic validation has been highlighted by previous studies [9,39]. Besides, most studies have focused on either postural ergonomic analysis or biomechanical analysis in their developed systems, while only a single study combined these two WMSD risk assessment methods [9]. The integration of these two methods could provide a more comprehensive interpretation of the assessed WMSD risk, making the system more attractive for industrial practitioners.

Therefore, this study aims to develop and validate a wearable inertial sensors-based automated system for evaluating WMSD risk in the workspace to support convenient and practical applications of on-site WMSDs assessment. We proposed a system to automatize the postural ergonomic analysis (RULA/REBA) and two-dimensional (2D) static biomechanical analysis based on the kinematic data from wearable inertial sensors, excluding the need for experts and complex data acquisition/processing. We further validated the accuracy of our system through a follow-up experiment with human subjects performing the representative tasks of different levels of difficulty to prove the robustness of our proposed system for evaluating tasks in various scenarios.

## 2. Materials and Methods

### 2.1. System Design

**Figure 1:**
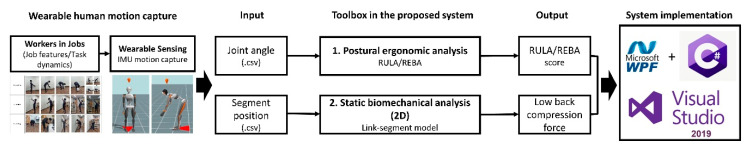
Conceptual design for the proposed system.

Figure 1 shows the conceptual design of the proposed system. The design specifications of the proposed wearable inertial sensors-based automated WMSD risk assessment system were determined by analyzing the functional requirements and needs of the industrial practitioners.

**Figure 2:**
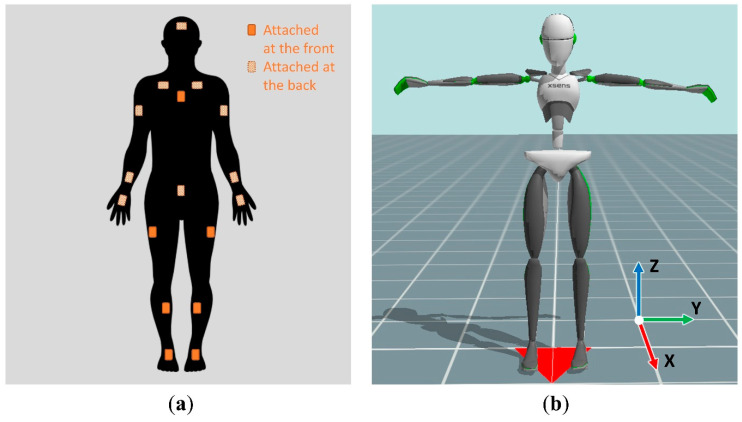
(**a**) Inertial sensor placement in the Xsens MVN Link motion capture system; (**b**) Global coordinates of the human body model built in the Xsens MVN Link.

In order to capture each worker’s full-body motions when performing the target task without interference on the task performance, 17 miniature inertial sensors were attached to the worker’s body. In this study, one commercially available wearable inertial sensors-based human motion capture system, the Xsens MVN Link (Xsens Technology BV, Enschede, Holland) [42,43], which has been widely used in the fields of ergonomics [25], biomechanics [44,45], and sports science [46,47] was used. Each inertial sensor in the Xsens MVN Link consisted of a 3D accelerometer, a 3D gyroscope, and a 3D magnetometer. The ranges for accelerometer, gyroscope and magnetometer were ±16 g, ±2000°/s and ±1.9 Gauss, respectively. These inertial sensors were equipped with the static accuracy of 0.2° for roll/pitch and 0.5° for yaw for orientation estimation. Figure 2 shows the sensor placement and the global coordinates of the human body model built in the Xsens system. The Xsens MVN Analyze software was used to create a kinematic model based on state-of-the-art miniature inertial sensors, sensor fusion algorithms and biomechanics models [42,43]. The performance analysis showed that the Xsens MVN Analyze could achieve root mean square error (RMSE) below 5° for the dominant joint angles during walking [43]. Kinematic information from the human motion data could be extracted using the Xsens MVN Analyze and converted to system input files including 3D angles for 22 skeletal joints and position data for 23 body segments with the time series. After importing the required input, the system could generate the assessment results automatically. The system was developed based on Windows Presentation Foundation with C# programming language; running in Windows operating system, thus, requires no any prerequisites for the hardware and software.

Our developed system is advantageous in terms of four aspects. First, the input of the system is generated by the inertial sensors-based motion capture system, which is applicable in the field environment unlike the optical motion capture system, while maintaining comparable accuracy [48]. Second, the system integrates postural ergonomic analysis and biomechanical analysis functions, which can provide a more comprehensive WMSD risk assessment results from different perspectives simultaneously. Third, the system presents the results with proper visualizations to aid quick interpretation and ergonomics intervention. It also provides an option to export the results for keeping the record or conducting further analysis. Lastly, the whole process can be done with a simple operation on the graphical user interface (GUI), which is easy and simple for industrial practitioners to use.

### 2.2. System Implementation

**Figure 3:**
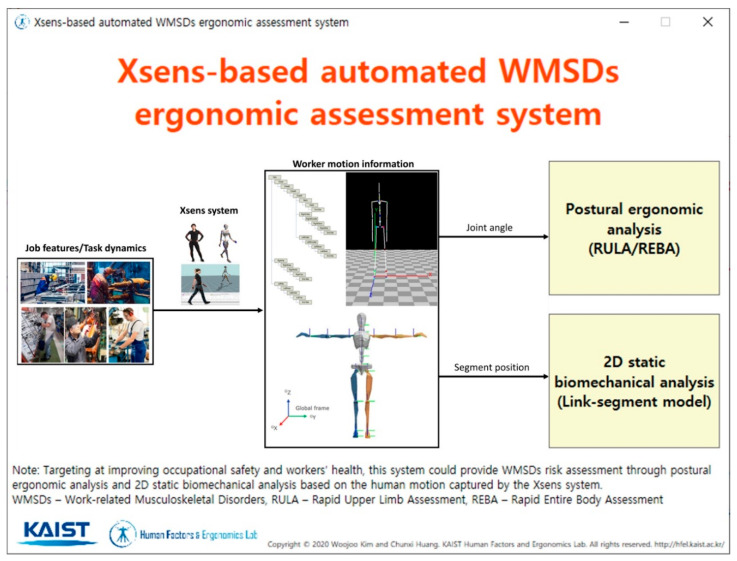
The main interface of the developed system.

Figure 3 shows the main interface of the developed system. The user could press the buttons located on the right to move onto either postural ergonomic analysis or static biomechanical analysis according to the user’s needs.

For the postural ergonomic analysis, the joint angle data were used to automatize the calculation of RULA/REBA scores based on the pre-defined thresholds and look-up tables. Required inputs related to task settings (force load, coupling, etc.) should be done manually by the analyst. RULA/REBA methods can provide an overview of WMSD risk on each body part by scoring based on the joint angle, and the grand scores can be determined based on the scores of each body part and some other factors (e.g., external load, coupling, and gravity-assistance) using look-up tables. The grand RULA/REBA scores are classified into different WMSD risk levels. RULA classifies the scores (range: 1–7) into the negligible risk (1–2), low risk (3–4), medium risk (5–6), and high risk (6+), and REBA classifies the scores (range: 1–15) into the negligible risk (1), low risk (2–3), medium risk (4–7), high risk (8–10), and very high risk (11+). Details about the RULA/REBA methods could refer to the original studies [19,20].

**Figure 4:**
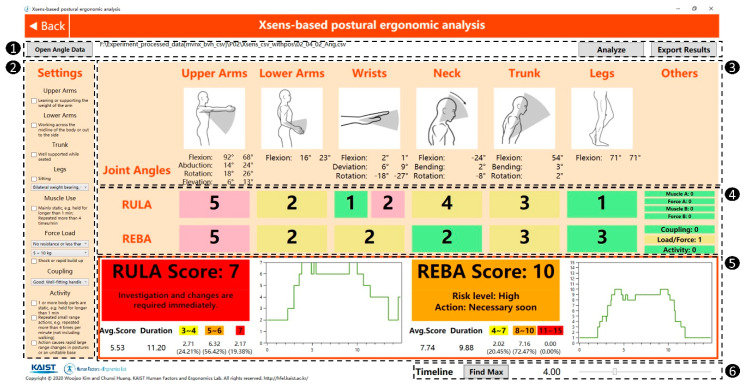
The GUI of the automated RULA/REBA assessment tool in the developed system. Numbers in black circles indicate the number of each section.

The GUI of the automated RULA/REBA assessment tool in the developed system is shown in Figure 4. The GUI of this tool is divided into 6 sections. After importing the joint angle data (Section 1) and manual inputs related to the task setting (Section 2), the analysis can be performed by clicking the “Analyze” button. The joint angles and corresponding sub-sectional scores per frame are presented in Sections 3 and 4, respectively, followed by the grand RULA/REBA scores with a standard set of colors to indicate different WMSD risk levels (Section 5). The average score, duration, and score distribution while performing the task are shown together to provide a better understanding of the WMSD risk. The time point can be freely adjusted with the slider located in Section 6 in Figure 4. Note that we also provide the “Find Max” function which can quickly adjust the time point to the moment with the highest RULA/REBA scores, as the users are generally more concerned with the worst case with the highest WMSD risk. All the assessment results can be exported and saved as a data file.

For the 2D static biomechanical analysis, the segment position data were used to automatize the calculation of low-back compression force and strength percent capable (%) of joints. The calculation is based on a 2D coplanar multiple-linkage static biomechanical model for symmetric sagittal plane activities [49]. The calculation of low-back compression force applying on the L5/S1 joint starts with the estimation of joint contact forces and moments, which can be calculated by summing the moments for each body segment in the linkage system. Using the moment at the L5/S1 joint, the abdominal pressure and the abdominal force are calculated based on empirical equations introduced in previous studies [50]. Since the compression and shear force at the L5/S1 joint are assumed to apply on the center of rotation of the disc, the muscle force on the spinal erector could be determined by the joint moment at L5/S1 and the abdominal force. Finally, the low-back compression force applying parallel to the disc compression force should be balanced. The static strength capabilities of all joints in the selected multiple-linkage model are calculated based on the population strength data from the 3D Static Strength Prediction Program (3DSSPP, Center for Ergonomics, University of Michigan). In our proposed system, the segment position data were projected into the sagittal plane to calculate the angles between segments of the multiple-linkage model.

**Figure 5:**
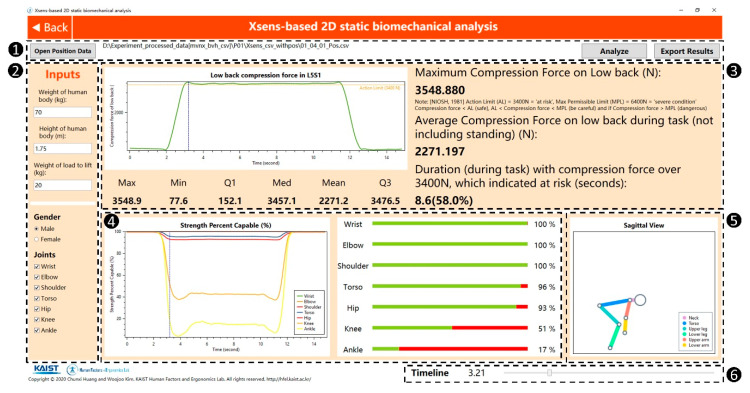
The GUI of the automated 2D static biomechanical analysis tool in the developed system. Numbers in black circles indicate the number of each section.

The GUI of the automated 2D static biomechanical analysis tool in the developed system is shown in Figure 5. The GUI of this tool can be divided into 6 sections likewise. Similar to the earlier case, Section 1 in Figure 5 accounts for input and output of the data, Section 2 accounts for manual settings, and Section 6 accounts for adjustment of the time point. After importing the segment position data (Section 1) and inserting the weight and height of the worker and the weight of the lifting load (Section 2), the analysis can be performed. The distribution and descriptive statistics of the low-back compression force are shown (Section 3), together with the strength percent capable (Section 4). Finally, the user can check the sagittal view of the worker’s posture represented as a link-segment model in Section 5.

### 2.3. System Validation

#### 2.3.1. Participants

For validation of the developed system, 20 young male subjects (age: 22.8 ± 2.0 years, height: 173.1 ± 4.8 cm, weight: 68.4 ± 7.4 kg, body mass index: 22.8 ± 2.1 kg/m^2^) participated in the experiment. All participants were healthy and without musculoskeletal diseases that may affect them performing experimental tasks. Each subject provided informed consent on the experimental protocol, which was approved by the university institutional review board (IRB-19-431).

#### 2.3.2. Experimental Procedures and Task Design

**Figure 6:**
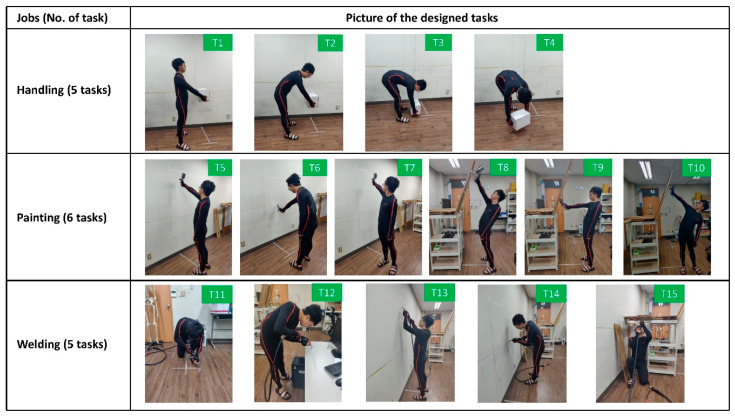
Designed tasks for validating the proposed system.

After the inertial sensors were attached to the body of participants, the system was calibrated to estimate the dimensions of the participant being tracked and the orientation of the sensors with respect to the corresponding body segments [43,51]. First, body height and foot length were input to apply dimensions of the participant on the human model. Second, participants were asked to hold the neutral upright standing pose with feet parallel pointing forward for around 4 s then walk forward, turn, and walk back to the original place in a normal pace. Lastly, participants were required to hold the neutral standing pose until the forward direction and origin of the local coordinate system was defined. Then, participants were instructed to conduct 15 experimental tasks (Figure 6) in randomized order. Fifteen experimental tasks derived from three common jobs in the shipbuilding process (manual handling, painting, and welding) were designed considering input from field experts of two shipbuilding factories. Four manual handling tasks (Task 1–4) with varying degrees of trunk flexion and twisting were designed, adapted from a study conducted in the manufacturing industry [26]. Participants were asked to handle a 5 kg external load in pre-defined positions. For the manual painting, two painting scenarios: painting on the vertical wall (Task 5–7) and painting on the non-vertical wall (with a slope of 25 degrees, task 8–10) were simulated based on [52]. Tasks within each painting scenario were varied in terms of painting direction (Task 5 and 8 for horizontal painting and Task 6 and 9 for vertical painting) and facing direction (Task 7 and 10 facing left while other tasks facing forward). The painting height, length of the painting path, and distance between the participant and wall were fixed across all painting tasks to minimize the potential confounding effects from other factors. For the manual welding, five tasks (Task 11–15) were designed considering the four basic welding positions (e.g., flat, horizontal, vertical, and overhead) and five main observed welding processes in the shipyard [53]. For painting and welding tasks, the painting gun and the welding gun models commonly used in the shipyard were held by the participants when performing tasks to simulate the real tasks in the field. Human motion data and videos of the participants while performing the designed tasks were recorded.

#### 2.3.3. Data Processing and Analysis

For validation of the RULA/REBA-based postural ergonomic analysis, the recorded task videos were delivered to two independent expert raters to obtain the reference RULA/REBA scores. The reliability of the system in estimating RULA/REBA scores was carried out using the intraclass correlation coefficient (ICC) [54,55], which is a widely used index in reliability analyses and represented by the ratio between variance of interest and total variance [56]. The interpretation of ICC follows previous guidelines [57]: ICC < 0.4, poor reliability; 0.4 < ICC < 0.59, fair reliability; 0.60 < ICC < 0.74, good reliability; ICC > 0.75, excellent reliability. The ICCs of RULA and REBA scores between two expert raters were 0.702 and 0.746, respectively, indicating good inter-rater reliability. Therefore, the average of RULA/REBA scores from two expert raters for each of the 15 tasks was compared with the RULA/REBA scores generated by our developed system. ICC and absolute difference between the RULA/REBA scores from the proposed system and experts’ ratings were calculated to check the degree of consistency and absolute error. The classification accuracy of WMSD risk levels based on RULA/REBA score ranges was also reported.

For validation of the 2D static biomechanical analysis, the low-back compression force estimated from the developed system was compared with the one calculated by 3DSSPP, one of the most widely used commercial programs for biomechanical analysis. As high low-back compression force is generally caused by the external load, four manual handling tasks (T1–T4) were analyzed for the validation of 2D static biomechanical analysis. The validity of the system in estimating low-back compression force was carried out using the intersystem coefficient of multiple correlation (CMC) [58,59,60,61], a frequently used dimensionless measure in the range [0, 1] to represent similarity of waveforms between systems. The interpretation of CMC follows previous guidelines [62]: CMC > 0.9, strong association; 0.5 < CMC < 0.9, moderate association; 0.25 < CMC < 0.5, weak association. Relative error in percentage (RMSE between predicted and reference values divided by the maximum reference value) was reported as well. SPSS Statistics 25 (SPSS Inc, Chicago, IL, USA) and R 3.5.3 (The R Foundation, Vienna, Austria) were used for conducting the statistical analysis.

## 3. Results

### 3.1. Validation of the RULA/REBA-Based Postural Ergonomic Analysis

Table 1 shows ICC of RULA/REBA scores between the developed system and the average scores of two expert raters were around 0.83 for both RULA and REBA methods, indicating excellent absolute agreement between the developed system and the expert raters. The mean absolute difference was lower than 0.5 and 1.0 for RULA and REBA, respectively. In addition, 95% of absolute differences were within 1.5 and 3.0 for RULA and REBA, respectively.

Table 2 and Table 3 show the RULA and REBA-based WMSD risk level classification agreement between the developed system and expert raters, respectively. Taking the experts’ ratings as a reference, the accuracy of our developed system for classifying a task into the right risk level is 88.3% (=(30 + 153 + 82)/300) for RULA, and 91.7% (=(18 + 191 + 66)/300) for REBA, which further suggests the effectiveness of our postural ergonomic analysis tool.

### 3.2. Validation of the 2D Static Biomechanical Analysis

**Figure 7:**
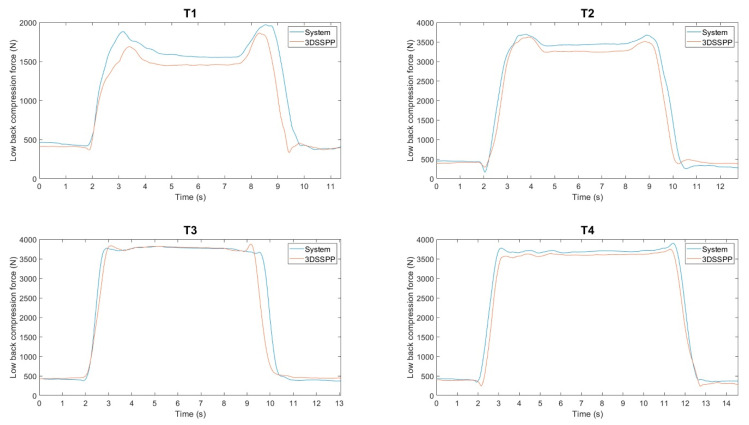
Low-back compression force of one participant acquired from the developed system and 3DSSPP for four manual handling tasks (T1–T4).

Figure 7 shows a representative example of low-back compression force from one participant acquired from the developed system and 3DSSPP for four manual handling tasks (T1–T4). The similar patterns indicate the good potential of our developed system in terms of static biomechanical analysis. To further quantify the agreement of the 2D low-back compression force between our developed system and reference system-3DSSPP, the CMC and relative error in percentages of the 2D low-back compression force between the developed system and 3DSSPP among 20 subjects are reported in Table 4. The high CMC values (>0.89) and small relative error in percentages (<9.5%) suggest good agreement between our system and 3DSSPP.

## 4. Discussion

### 4.1. System Development

Our proposed system integrated the RULA/REBA-based postural ergonomic analysis and 2D static biomechanical analysis to enable a comprehensive WMSD risk assessment in the workspace. RULA/REBA can provide a global assessment of WMSD risk based on simple principles [63], and offer general guidance based on the overall risk level. However, RULA and REBA oversimplify the assessment of some critical anatomical body segments such as the low back which plays an important role in inducing WMSD [64,65]. Our system can further assess the WMSD risk caused by the low back by calculating the low-back compression force, a very important and useful indicator of low-back risk [66], through the 2D static biomechanical analysis. Interestingly, only one study from Golabchi et al. proposed an automated WMSD assessment system that combined the RULA-based postural ergonomic analysis and the static biomechanical analysis [9]. There are two major differences between our proposed system and Golabchi’s system. First, we used a wearable inertial sensors-based motion capture system for obtaining kinematic input while they extracted the human motion data from a virtual human body model built through simulation [67] or manually. The wearable inertial sensors were proposed in this study for reliable and convenient real-time in-situ data acquisition in the workspace [57]. Second, we developed our own 2D static biomechanical analysis tool while their system relied on the commercial software (3DSSPP). Given the higher accuracy of human motion capture and the ease of input preparation, our system could be valuable for the WMSDs risk assessment in the industrial workspace.

Our RULA/REBA-based postural ergonomic assessment tool provides several attractive features compared to similar systems reported in previous studies [38,40]. It can provide not only the grand RULA/REBA score and corresponding color-coded WMSD risk level, but also real-time sub-sectional scores for each body segment, which can help to broaden understanding and locate the potentially problematic body segment to gain more insights for preventing WMSDs. In addition, the RULA and REBA scores are calculated and visualized for each time point during the task motion together with extra descriptive statistics (average score, duration, and risk distribution while performing the task) to provide more comprehensive information about the WMSD risk of specific tasks, by extending the indicators used in traditional RULA/REBA methods. The user is allowed to check the grand RULA/REBA and sub-sectional scores of any time points by freely controlling the slider. Lastly, the maximum RULA/REBA scores representing the highest overall WMSD risk during the task can be found by clicking the “Find Max” button, which helps the user to easily find the moment with the most awkward posture within a short time.

In this study, we also developed the 2D static biomechanical analysis tool based on a 2D multi-linkage model of the human body, without typical complicated data preprocessing for biomechanical analysis of WMSD risk assessment as in previous studies [9,31,68]. Despite its simplicity, the low-back compression force calculated by the automated 2D static biomechanical analysis added in our system is of great importance in indicating the WMSD risk on the low back when the workers lift various loads [49]. Existing studies mainly relied on the commercial software 3DSSPP for their static biomechanical analysis [9,31]. Compared with the 3DSSPP, our proposed system has fewer functions but focused on two core measurements of the 2D static biomechanical analysis: 2D low-back compression force and strength percent capable (%). Our system provides graphs showing an overview of low-back compression force and static strength percentile during the whole task. Graphs can help the user to easily visualize the changes and identify the riskiest time point, which could be a useful function but not enabled in 3DSSPP. Moreover, it allows the user to check the corresponding assessment results at different time points easily using a slider, which is more convenient than the 3DSSPP especially when analyzing long tasks.

### 4.2. System Validation

Most of the previous studies have not validated the accuracy of their proposed systems. Even for a few studies that conducted validation experiments, the researchers only included simple experimental tasks such as bending, scanning, or cutting [9,40,41]. To validate the accuracy of our developed system, a follow-up experiment including representative jobs and tasks with different levels of difficulty was designed and conducted.

According to our experimental results, the developed system can generate accurate and reliable RULA/REBA scores. The scores generated from our system had high accuracy for both RULA and REBA methods (88.3% for RULA and 91.7% for REBA) when compared to the scores from the expert raters, which outperformed the accuracy of 83.3% using RULA reported by a previous study [9]. One possible reason for this is that the results were highly influenced by the accuracy of captured human motion data, as we used an inertial sensors-based motion capture system while they used virtual human body models built from simulation or manually. Another previous study reported a higher accuracy of 94.79% for the RULA assessment method [40], however, it was only validated against a simple scanning task, not tasks with different levels of difficulty. Meanwhile, we also validated the 2D static biomechanical analysis tool in our developed system by comparing the low-back compression force generated from our system and 3DSSPP. Results indicated that the developed system has good accuracy (mean CMC of ~0.9 compared with the reference system and mean relative error in a percentage ranging from 3.42% to 9.34%) and convenience for calculating low-back compression force on the sagittal plane.

An interesting point about the use of ICC for reliability assessment should be noted. A previous study [69] showed that the ICC estimate can be influenced by not only the rater quality, but also distribution of subjects. Their simulation results suggested that ICC from the convex or skewed distribution could be underestimated compared to ICC from the uniform distribution. In the present study, it was operationally challenging to enroll a uniform distribution of subjects across grades, and the RULA scores were left skewed while the REBA scores followed the convex distribution. Therefore, there is a chance that reported ICCs were underestimated, despite the fact that the reported ICCs already showed excellent reliability of the developed system. It is expected that the majority of tasks leaned towards the high RULA scores, since experimental tasks derived from the shipbuilding process would naturally include awkward postures with high WMSD risks. These tasks mainly burdened upper limbs thus skewness could appear higher on RULA than on REBA.

### 4.3. Limitations and Future Work

Several limitations of this study should be noted. First, the developed system relies on a commercial inertial sensors-based human motion capture system to acquire the accurate kinematic input (e.g., joint angle and segment position) necessary for the assessment of WMSD risk. Even though the assessment can be done with the same type of input data acquired from other motion capture systems, the validity of the results could be dependent on the accuracy of inputs. Future work could validate the system with kinematic data directly acquired from a set of low-cost inertial sensors based on sensor fusion algorithms. Second, for the development of the static biomechanical analysis tool, we used a simplified 2D coplanar multiple-linkage static biomechanical model for symmetric sagittal plane activities, which is less accurate than the 3D models for certain tasks involving twists. Future work could perform static biomechanical analysis using a 3D multiple-linkage model. Last but not least, the validation experiment was conducted in the lab environment with participants using convenience sampling instead of in the field environment with industrial workers due to various constraints. To validate the practicality and applicability of the developed system, field experiments in the real workplace with workers could be conducted further.

## 5. Conclusions

We developed a wearable inertial sensors-based automated system for assessing WMSD risk in the workspace. Miniature inertial sensors can be worn by the worker to capture human motions accurately and conveniently and collected kinematic data are used as an input of the developed system. The system is equipped with a user-friendly GUI, showing the results of WMSD risk assessment based on postural ergonomic analysis in terms of RULA/REBA methods and static biomechanical analysis in terms of 2D low-back compression force and strength percent capability. The accuracy of the proposed system was validated through a follow-up experiment among 20 subjects when performing representative tasks in the heavy industry. Results showed that the RULA/REBA scores and low-back compression force derived from our system achieved high agreement with the expert raters (ICC ≥0.83, classification accuracy >88%) and reference system (mean intersystem CMC >0.89 and relative error <9.5%), respectively. The findings suggested that the developed wearable inertial sensors-based automated system could be effectively used for WMSD risk assessment of workers when performing tasks in the workspace.

## Figures and Tables

**Table 1:** Intraclass correlation coefficient (ICC) and absolute difference of RULA/REBA scores between the developed system and the expert raters.

Postural Ergonomic Assessment	ICC		Absolute Difference	
	Coefficient	95% CI	Mean ± SD	95% CI
RULA	0.836	(0.757, 0.885)	0.455 ± 0.418	(0, 1.5)
REBA	0.830	(0.736, 0.885)	0.923 ± 0.774	(0, 3.0)

**Table 2:** RULA-based WMSD risk level classification agreement between the developed system and the expert raters.

RULA-Based WMSD Risk Level		Expert Raters			Total
		Low Risk	Medium Risk	High Risk	
**The developed system**	**Low risk**	30	8	0	38
	**Medium risk**	2	153	15	170
	**High risk**	0	10	82	92
**Total**		32	171	97	300

**Table 3:** REBA-based WMSDs risk level classification agreement between the developed system and the expert raters.

REBA-Based WMSD Risk Level		Expert Raters			Total
		Low Risk	Medium Risk	High Risk	
**The developed system**	**Low risk**	18	1	0	19
	**Medium risk**	1	191	12	204
	**High risk**	0	11	66	77
**Total**		19	203	78	300

**Table 4:** Coefficient of multiple correlation (CMC) and relative error in percentages between the 2D low-back compression force (unit: Newton) generated from the developed system and 3DSSPP for four selected manual handling tasks (T1–T4).

Task	T1	T2	T3	T4
CMC (mean ± SD)	0.896 ± 0.029	0.902 ± 0.031	0.923 ± 0.026	0.927 ± 0.027
Relative error in percentage (mean ± SD)	9.34% ± 2.19%	3.42% ± 0.58%	4.19% ± 0.46%	3.91% ± 0.45%
